# Free Vibration Behavior of CFRP Composite Sandwich Open Circular Cylindrical Shells with 3D Reentrant Negative Poisson’s Ratio Core

**DOI:** 10.3390/polym17172276

**Published:** 2025-08-22

**Authors:** Shi-Chen Liu, Yun-Long Chen

**Affiliations:** School of Aerospace Engineering, Xiamen University, Xiamen 361005, China

**Keywords:** CFRP composite, sandwich cylindrical shell, free vibration, negative Poisson’s ratio core, analytical model

## Abstract

This study explores the free vibration behavior of carbon fiber-reinforced sandwich open circular cylindrical shells featuring 3D reentrant auxetic cores (3D RSOCCSs). For theoretical predictions, a model integrating the Rayleigh–Ritz method (RRM) and Reddy’s third-order shear deformation theory (TOSDT) is adopted, whereas the finite element analysis approach is used for simulation predictions. All-composite 3D RSOCCSs specimens are produced via hot-press molding and interlocking assembly, and the modal characteristics of 3D RSOCCSs are obtained through hammer excitation modal tests. The predicted modal properties are in good agreement with the experimental results. In addition, the influences of fiber ply angles and geometric parameters on the natural frequency in the free vibration are thoroughly analyzed, which can offer insights for the vibration analysis of lightweight auxetic metamaterial cylindrical shells and promote their practical use in engineering scenarios focused on vibration mitigation.

## 1. Introduction

Materials and structures characterized by auxetic behavior—defined by a negative Poisson’s ratio (NPR)—are often referred to as anti-rubber materials or those with dilatational properties [1]. This unique class of materials stands in stark contrast to conventional materials, which typically exhibit a positive Poisson’s ratio; when subjected to axial stretching, auxetics undergo transverse elongation rather than contraction, and conversely, they contract transversely under compression [2,3]. This counterintuitive deformation mechanism, observable across scales from the microscopic (e.g., molecular lattices) to the macroscopic (e.g., engineered structures) [2,4], distinguishes auxetics fundamentally from their conventional counterparts [5,6].

The rapid advancement of 3D additive manufacturing technologies has been a transformative force in the field of auxetic metamaterials [7,8,9]. These technologies have overcome longstanding fabrication barriers, enabling the production of complex periodic cellular structures with precisely controlled geometries—such as re-entrant honeycombs, chiral lattices, and hierarchical trusses, arrowhead-on cores [10], triangular chiral (Tri-Chi) honeycombs [11], three-dimensional augmented re-entrant cellular structures (3D-ARCS) [12], and butterfly shaped honeycombs [13]—that were previously unfeasible with traditional manufacturing methods [14,15]. This technological leap has catalyzed systematic investigations into the fundamental mechanical properties of auxetic systems, with a large body of literature [16,17,18,19,20,21] documenting studies on topological optimization of unit cells, quantification of Poisson’s ratio across deformation ranges, and modeling of equivalent stiffness for engineering design. Researchers have explored diverse unit cell configurations, each tailored to achieve specific performance trade-offs: for example, re-entrant honeycombs prioritize high NPR magnitudes, while chiral lattices offer a balance between flexibility and load-bearing capacity [8,22]. And Tri-Chi honeycombs combine honeycomb and chiral features to tune Poisson’s ratio and stiffness [11].

Recent years have seen auxetic metamaterials research shift to multifunctional design, focusing on free vibration. This focus on vibration has spurred investigations across diverse structural forms and loading scenarios. For random vibration, studies have developed equivalent models (e.g., variational asymptotic method-based models for arrowhead-on core sandwich plates [10], 2D Reissner–Mindlin models for Tri-Chi honeycomb panel [11]) to efficiently predict power spectral density (PSD) and root mean square (RMS) responses, revealing that core geometry (e.g., ligament-rib angle) and face sheet layup significantly influence dynamic performance. Free vibration analyses have spanned structures from plates (star-shaped auxetic rectangular plates [23], sandwich plates with inverse-designed 3D auxetic cores [24], hygrothermal environment-exposed sandwich plates with GPLRC face sheets [25]) to shells (conical sandwich shells with functionally graded auxetic honeycomb cores [26], doubly curved butterfly shaped auxetic nanoshells [13], stiffened doubly curved sandwich shells [27]) and beams (axially loaded super-light auxetic beams with FG face sheets [28]). These studies employ a range of theoretical frameworks, including classical plate theory (CPT) [23], first-order shear deformation theory (FSDT) [26,28,29], Reddy’s third-order shear deformation theory (TSDT) [25,30], and nonlocal strain gradient theory (NSGT) [13], often paired with methods like the Rayleigh–Ritz [23,30], differential quadrature (DQM) [30], and multiple-scale technique [28,29].

Nonlinear vibration behavior has also garnered attention, with research on FG auxetic cylinders [29] and axially loaded beams [28] revealing frequency-amplitude relationships and hardening/softening characteristics dependent on material gradation and boundary conditions. Additionally, specialized scenarios—such as vibration in liquid-filled shells [30], viscoelastic foundations [31], and structures with cutouts [32]—have been explored, highlighting the role of environmental interactions and geometric features in dynamic response. For example, studies on liquid-filled functionally graded auxetic sandwich shells show that liquid level and core geometric parameters strongly affect natural frequencies [30], while viscoelastic foundation models reveal that damping coefficients and auxetic core properties synergistically influence visco-vibration behavior [31]. Vibration-related multifunctionality is another active area, including energy harvesting via arc-shaped auxetic cantilever beams [8] and vibroacoustic control in novel auxetic honeycomb sandwich panels with polyurea-metal laminate face sheets [33]. The latter demonstrates that symmetrical face sheet structures and optimized honeycomb tilt angles (e.g., 45°) can reduce vibration peaks and improve sound insulation by up to 9.4% [33], underscoring the potential of auxetics in noise mitigation.

Inspired by natural cellular architectures—such as the hierarchical, load-adaptive structures found in wood (with radial-axial cell alignment), bone (trabecular lattices), and avian beaks (gradient porosity)—engineered cellular systems like honeycombs and lattice trusses have been widely adopted in aerospace, automotive, and civil engineering for their lightweight and high-strength characteristics [34]. Parallel to this evolution, research on negative Poisson’s ratio materials has progressed from early studies on disordered foam structures [35] to the design of precisely engineered periodic honeycomb configurations with tailored NPR values [36]. Within this context, Scarpa et al. contributed foundational work by applying first-order plate theory to determine the fundamental frequencies of auxetic sandwich laminates under cylindrical bending conditions [37], while finite element models of microstructures have enabled detailed analyses of static and viscoelastic behaviors in closed-cell re-entrant honeycombs with viscoelastic infills [38]. Advanced experimental tools, such as scanning laser vibrometry, have been paired with full-scale finite element simulations to validate modal analysis results, including dynamic responses of auxetic gradient honeycomb composites [39]. Studies on anti-tetra chiral honeycomb panels incorporating metal rubber particles have further confirmed the feasibility of designing integrated auxetic structures that balance lightweight design with robust damping and load-bearing capabilities [40]. Recent work has also extended to aeroelastic stability, with analyses of ring-stiffened conical sandwich shells with FG auxetic core [41] showing that optimal ring placement enhances critical aerodynamic pressure, bridging vibration dynamics and structural stability.

Recent advances by Duc et al. have significantly expanded this body of knowledge through the development of nonlinear governing equations for auxetic honeycomb-cored sandwich structures. Leveraging first-order shear deformation theory combined with von Kármán geometric nonlinearity, and integrating analytical methods such as the Airy stress function, Galerkin technique, and fourth-order Runge–Kutta method, their work has addressed dynamic responses in diverse structural configurations: sandwich panels [42], magneto-electro-elastic face sheet laminates [43], double-curved shallow shells [44,45], composite cylindrical shells [46], and stiffened circular cylindrical shells [47]. While these findings provide valuable insights for the design of auxetic composites under various mechanical loads [48,49,50,51], understanding of their intrinsic vibration and damping mechanisms—particularly how NPR influences energy dissipation, modal coupling, and frequency response—remains in its early stages. This gap is further underscored by the need for more research on specific configurations, such as sandwich cylindrical shells with auxetic truss cores, which are less explored compared to honeycomb or chiral cores [52,53,54].

Sandwich cylindrical shell structures [55], valued for their exceptional design flexibility, high specific stiffness, and ability to withstand complex loading environments [56], have seen growing adoption in demanding engineering fields [57], including aerospace (e.g., rocket fairings), marine (e.g., submersible hulls), and high-speed rail (e.g., bogie enclosures) [58]. Lakes’ structural hierarchy framework highlighted that composite systems can simultaneously achieve superior stiffness and vibration performance [59,60], underscoring the potential of integrating the high mechanical capacities of composite materials with the tunable vibrational properties of auxetic structures in sandwich cylindrical shells. This synergistic approach offers a promising pathway for vibration mitigation in critical engineering systems.

However, despite extensive research on auxetic materials and sandwich structures, significant gaps remain in the current literature, particularly regarding the free vibration characteristics of composite sandwich structures with auxetic truss cores, and critical gaps persist in understanding the dynamic behavior of open cylindrical shells integrated with 3D reentrant auxetic cores—a configuration that merges the curved geometry of cylindrical shells, the lightweight advantage of sandwich structures, and the unique deformation mechanism of 3D auxetic cores. Specifically, existing studies on auxetic-cored sandwich structures have predominantly focused on planar panels or closed cylindrical shells, overlooking open circular cylindrical shells that are critical for applications like rocket fairings and submersible hulls. Most investigations have also relied on 2D auxetic cores, with scarce experimental validation for all-composite 3D reentrant auxetic-cored shells. Further, prior research has centered on: (1) auxetic flat plates, where boundary conditions and deformation modes differ fundamentally from curved shells; (2) closed cylindrical shells with 2D auxetic cores, which lack the stiffness asymmetry introduced by open boundaries; and (3) 3D auxetic structures with simple geometries, without coupling to composite laminates. Consequently, the free vibration characteristics of 3D reentrant auxetic core-reinforced CFRP sandwich open cylindrical shells (3D RSOCCSs)—including their modal patterns, frequency responses, and sensitivity to design parameters—remain uncharacterized. The present study aims to address these gaps through a systematic, multi-method investigation (theoretical, numerical, and experimental) of the free vibration of 3D reentrant auxetic-cored open cylindrical shells, laying the foundation for understanding and engineering such complex structures.

This paper focuses on investigating the free vibration properties of carbon-fiber-reinforced sandwich open circular cylindrical shells with a 3D re-entrant auxetic core. To forecast the modal characteristics of this structure, a Rayleigh–Ritz vibration model is established based on the Reddy third-order shear deformation theory. Then, all-composite sandwich shell samples are produced through a combination of hot-press molding and interlocking assembly techniques. Modal tests are utilized to evaluate the vibration properties of these structures. Subsequently, a finite element model is applied to assess the structural performance of free vibration. Finally, a comprehensive parametric study is performed to delineate the influence of design variables on the natural frequencies of the structure.

## 2. Theoretical Formulations

### 2.1. Assumption and Modeling

**Figure 1:**
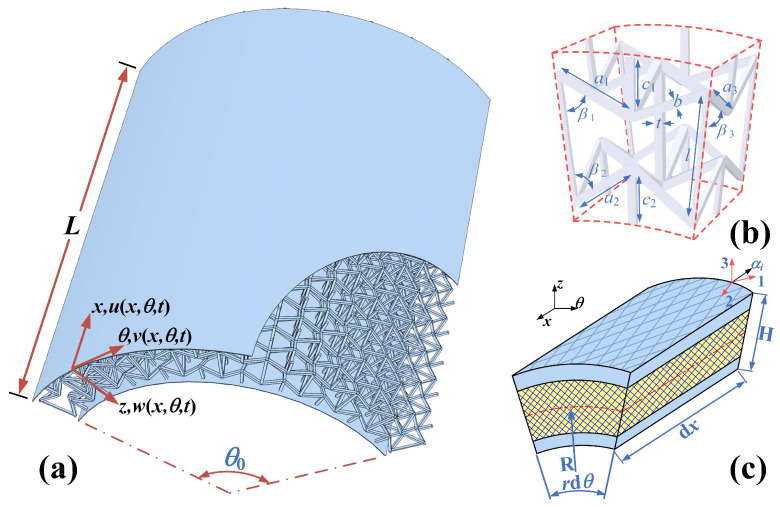
Schematic illustration of (**a**) 3D RSOCCSs, (**b**) geometric configuration and coordinate system, (**c**) representative unit cell.

Figure 1a presents the structural model of 3D RSOCCSs, comprising composite laminate shells, a 3D re-entrant auxetic core, and damping laminates. Herein, the symbols ‘L’, ‘R’, ‘$\theta_{0}$’, and ‘h’ denote length, radius, circumferential angle, and thickness, respectively. A representative sandwich shell unit with lengths dx and rdθ is illustrated in Figure 1b, where the global coordinate system of the structure is established on the mid-plane as shown in Figure 1a. The primary material coordinate system of the composite is denoted by the indices 1, 2, and 3 in Figure 1b, while the 3D re-entrant auxetic core units are depicted in Figure 1c.

Furthermore, the following presumptions require prior clarification:

(1) The thickness of ideal interfacial bonds between components is negligible, meaning the deformation across layers remains continuous;

(2) Relative to its dimensions, the structural deformation is minimal, adhering to the “small deformation hypothesis”;

(3) All constituent parts are fabricated from linear elastic materials, following the “linear elasticity assumption”;

(4) Normal strains perpendicular to the mid-plane are negligible.

### 2.2. Equivalent Modulus of Re-Entrant Truss Core

Based on the analytical approach for predicting the effective stiffness of re-entrant honeycombs [34], the effective modulus of the 3D re-entrant auxetic core can be derived in a comparable manner, expressed as follows:

(1) $$E_{1}^{c}=\frac{4(l-a\sin\beta )}{a^{2}\cos^{2}\beta }\frac{K_{f}^{a}K_{s}^{a}K_{s}^{l}}{K_{f}^{a}K_{s}^{a}+16K_{s}^{a}K_{s}^{l}\Theta \cos^{2}\beta +16K_{f}^{a}K_{s}^{l}\Theta \sin^{2}\beta }$$

(2) $$E_{2}^{c}=\frac{a\cos\beta }{l^{2}}\frac{K_{s}^{a}K_{f}^{a}}{K_{f}^{a}-\Theta \prime \sin\beta (K_{f}^{a}+K_{s}^{a})}$$

(3) $$G_{12}^{c}={\left[\frac{1}{K_{f}^{l}}\frac{2a\cos\beta }{l-a\sin\beta }+\frac{1}{K_{f}^{a}}(\frac{1}{a}-\frac{\sin\beta }{2l-2a\sin\beta })(l\cos\beta +2a\sin\beta )\right]}^{-1}$$

(4) $$G_{23}^{c}=\frac{K_{f}^{a}}{2l-2a\sin\beta }$$

(5) $$v_{12}^{c}=-\frac{\left(l-a\sin\beta \right)}{a\cos\beta }\frac{16\Theta K_{s}^{l}(K_{s}^{a}\sin\beta -K_{f}^{a}\tan\beta )}{K_{f}^{a}K_{s}^{a}+16\Theta K_{s}^{a}K_{s}^{l}\cos\beta +16\Theta K_{f}^{a}K_{s}^{l}\tan\beta \sin\beta }$$

where $K_{s}^{a}$ and $K_{s}^{l}$ denote the tensile force constants of struts with lengths a and l, respectively. $K_{f}^{a}$ represents the flexural force constant of struts with length a. The specific expressions for $K_{s}^{a}$, $K_{s}^{l}$, $K_{f}^{a}$, Θ and Θ′ are given in the Appendix A.

### 2.3. Kinematic and Stress–Strain Relations

Using the Reddy third-order shear deformation theory [61], the displacement components u, v and w at a point (x,θ,z) within the sandwich open laminated cylindrical shell are formulated as

(6) $$\begin{array}{l} {\tilde{u}}^{k}(x,\theta ,z^{k},t)=u^{k}(x,\theta ,t)+f(z^{k})\frac{\partial w}{\partial x}+g(z^{k})\vartheta_{1}^{k}(x,\theta ,t)\\ {\tilde{v}}^{k}(x,\theta ,z^{k},t)=(1+\frac{z^{k}}{R^{k}})v^{k}(x,\theta ,t)+f(z^{k})\frac{1}{R^{k}}\frac{\partial w}{\partial \theta }+g(z^{k})\vartheta_{2}^{k}(x,\theta ,t)\\ {\tilde{w}}^{k}(x,\theta ,z^{k},t)=w(x,\theta ,t) \end{array}$$

where t denotes the time variable; ${\tilde{u}}^{\left(i\right)}$, ${\tilde{v}}^{\left(i\right)}$, and ${\tilde{w}}^{\left(i\right)}$ represent the generalized displacements of the *i*-th layer in the x, θ, and z directions, respectively. Meanwhile, $u^{\left(i\right)}$ and $v^{\left(i\right)}$ signify the mid-plane displacements in the x and θ directions, correspondingly. Additionally, $\vartheta_{1}^{\left(i\right)}$ and $\vartheta_{2}^{\left(i\right)}$ denote the rotations of the transverse normal to the mid-plane about the circumferential and axial coordinates, respectively. Here, $z^{\left(i\right)}\in \left[-h_{i}/2,h_{i}/2\right]$ is measured from the mid-plane of the *i*-th layer. Both $f(z)=-4z^{3}/2h^{2}$ and $g(z)=z-4z^{3}/2h^{2}$ characterize the displacement distribution along the z direction and are referred to as the generalized displacement distribution shape functions.

The relationships between strain and displacement are expressed as follows

(7) $$\begin{array}{l} \varepsilon_{x}^{k}=\frac{\partial u^{k}}{\partial x}+f\frac{\partial^{2}w^{k}}{\partial x^{2}}+g\frac{\partial \varphi_{x}^{k}}{\partial x}\\ \varepsilon_{\theta }^{k}=\frac{1}{R}(\frac{\partial v^{k}}{\partial \theta }+w^{k})+z\frac{1}{R^{2}}\frac{\partial v^{k}}{\partial \theta }+f\frac{1}{R^{2}}\frac{\partial^{2}w^{k}}{\partial \theta^{2}}+g\frac{1}{R}\frac{\partial \varphi_{\theta }^{k}}{\partial \theta }\\ \gamma_{x\theta }^{k}=\frac{1}{R}\frac{\partial u^{k}}{\partial \theta }+\frac{\partial v^{k}}{\partial x}+z\frac{1}{R}\frac{\partial v^{k}}{\partial x}+f\frac{2}{R}\frac{\partial^{2}w^{k}}{\partial x\partial \theta }+g(\frac{1}{R}\frac{\partial \varphi_{x}^{k}}{\partial \theta }+\frac{\partial \varphi_{\theta }^{k}}{\partial x})\\ \gamma_{xz}^{k}=(1+\frac{\partial f}{\partial z})\frac{\partial w^{k}}{\partial x}+\frac{\partial g}{\partial z}\varphi_{x}^{k}\\ \gamma_{\theta z}^{k}=-f\frac{1}{R^{2}}\frac{\partial w^{k}}{\partial \theta }-g\frac{1}{R}\varphi_{\theta }^{k}+(1+\frac{\partial f}{\partial z})\frac{1}{R}\frac{\partial w^{k}}{\partial \theta }+\frac{\partial g}{\partial z}\varphi_{\theta }^{k} \end{array}$$

where $\varepsilon_{x}^{k},\varepsilon_{\theta }^{k},\gamma_{x\theta }^{k},\gamma_{\theta z}^{k},\gamma_{zx}^{k}$ denote the normal strain components and shear strain components within the mid-plane of the *k*-th layer, respectively.

The relevant stresses σ and τ within the *k*-th layer may be computed via the generalized Hooke’s law, expressed as

(8) $$\left\{\begin{array}{c} \sigma_{x}^{k}\\ \sigma_{\theta }^{k}\\ \tau_{x\theta }^{k}\\ \tau_{xz}^{k}\\ \tau_{\theta z}^{k} \end{array}\right\}=\left\{\begin{array}{ccccc} {\bar{\tilde{Q}}}_{11}^{k} & {\bar{\tilde{Q}}}_{12}^{k} & {\bar{\tilde{Q}}}_{16}^{k} & 0 & 0\\ {\bar{\tilde{Q}}}_{12}^{k} & {\bar{\tilde{Q}}}_{22}^{k} & {\bar{\tilde{Q}}}_{26}^{k} & 0 & 0\\ {\bar{\tilde{Q}}}_{13}^{k} & {\bar{\tilde{Q}}}_{26}^{k} & {\bar{\tilde{Q}}}_{66}^{k} & 0 & 0\\ 0 & 0 & 0 & {\bar{\tilde{Q}}}_{44}^{k} & {\bar{\tilde{Q}}}_{45}^{k}\\ 0 & 0 & 0 & {\bar{\tilde{Q}}}_{45}^{k} & {\bar{\tilde{Q}}}_{55}^{k} \end{array}\right\}\left\{\begin{array}{c} \varepsilon_{x}^{k}\\ \varepsilon_{\theta }^{k}\\ \gamma_{x\theta }^{k}\\ \gamma_{xz}^{k}\\ \gamma_{\theta z}^{k} \end{array}\right\}$$

where $\left(\sigma_{x}^{k},\sigma_{\theta }^{k}\right)$ and $\left(\varepsilon_{x}^{k},\varepsilon_{\theta }^{k}\right)$ represent the normal stresses and the corresponding normal strain of the *k*-th layer along the principal coordinate axis x and θ. For shear -related quantities, $\left(\tau_{x\theta }^{k},\tau_{xz}^{k},\tau_{\theta z}^{k}\right)$ and $\left(\gamma_{x\theta }^{k},\gamma_{xz}^{k},\gamma_{\theta z}^{k}\right)$ indicate the shear stresses and shear strain within the *k*-th layer along the principal coordinate plane xθ, xz and θz. The elastic constants matrix $\tilde{Q}$ under the principal coordinate system are expressed via the following equations:

(9) $$\left[\begin{array}{ccccc} {\tilde{Q}}_{11}^{k} & {\tilde{Q}}_{12}^{k} & {\tilde{Q}}_{16}^{k} & 0 & 0\\ {\tilde{Q}}_{12}^{k} & {\tilde{Q}}_{22}^{k} & {\tilde{Q}}_{26}^{k} & 0 & 0\\ {\tilde{Q}}_{16}^{k} & {\tilde{Q}}_{26}^{k} & {\tilde{Q}}_{66}^{k} & 0 & 0\\ 0 & 0 & 0 & {\tilde{Q}}_{44}^{k} & {\tilde{Q}}_{45}^{k}\\ 0 & 0 & 0 & {\tilde{Q}}_{45}^{k} & {\tilde{Q}}_{55}^{k} \end{array}\right]=T\left[\begin{array}{ccccc} Q_{11}^{k} & Q_{12}^{k} & Q_{16}^{k} & 0 & 0\\ Q_{12}^{k} & Q_{22}^{k} & Q_{26}^{k} & 0 & 0\\ Q_{16}^{k} & Q_{26}^{k} & Q_{66}^{k} & 0 & 0\\ 0 & 0 & 0 & Q_{44}^{k} & 0\\ 0 & 0 & 0 & 0 & Q_{55}^{k} \end{array}\right]T^{T}\;T=\left[\begin{array}{ccccc} \cos^{2}\alpha^{k} & \sin^{2}\alpha^{k} & -2\cos\alpha^{k}\sin\alpha^{k} & 0 & 0\\ \sin^{2}\alpha^{k} & \cos^{2}\alpha^{k} & 2\cos\alpha^{k}\sin\alpha^{k} & 0 & 0\\ \cos\alpha^{k}\sin\alpha^{k} & -\cos\alpha^{k}\sin\alpha^{k} & \cos^{2}\alpha^{k}-\sin^{2}\alpha^{k} & 0 & 0\\ 0 & 0 & 0 & \cos\alpha^{k} & \sin\alpha^{k}\\ 0 & 0 & 0 & -\sin\alpha^{k} & \cos\alpha^{k} \end{array}\right]$$

(10) $$\begin{array}{l} Q_{11}^{k}=\frac{E_{11}^{k}}{1-\mu_{12}^{k}\mu_{21}^{k}},Q_{44}^{k}=G_{23,}^{k}\\ Q_{12}^{k}=\frac{\mu_{12}^{k}E_{22}^{k}}{1-\mu_{12}^{k}\mu_{21}^{k}},Q_{55}^{k}=G_{13,}^{k}\\ Q_{22}^{k}=\frac{E_{22}^{k}}{1-\mu_{12}^{k}\mu_{21}^{k}},Q_{66}^{k}=G_{12}^{k} \end{array}$$

where $\cos\alpha^{k}$ and $\sin\alpha^{k}$ denote the directional coefficients within the *k*-th layer, where $\alpha^{k}$ corresponds to the angle between the principal coordinate system and the principal direction of the fiber. Additionally, $Q_{ij}^{k}(i,j=1,2,3,4,5,6)$ represents the elastic constants in the material coordinate system, while $E_{11}^{k},E_{22}^{k},G_{12}^{k},G_{23}^{k},G_{13}^{k}$ and $\mu_{12}^{k},\mu_{21}^{k}$ stand for the engineering parameters of the *k*-th layer.

### 2.4. Energy Expressions

The Rayleigh–Ritz method, focusing on energy principles, is employed in this study. This selection is driven by the consistency of its results and the efficiency in its modeling and solution processes. For the 3D RSOCCSs, the total strain energy U and the kinetic energy T are expressed as

(11) $$U=\frac{1}{2}\sum_{k=1}^{K}\int_{-h_{k}/2}^{h_{k}/2}\int_{0}^{\theta_{0}}\int_{0}^{L}(\sigma_{x}^{k}\varepsilon_{x}^{k}+\sigma_{\theta }^{k}\varepsilon_{\theta }^{k}+\tau_{x\theta }^{k}\gamma_{x\theta }^{k}+\tau_{\theta z}^{k}\gamma_{\theta z}^{k}+\tau_{zx}^{k}\gamma_{zx}^{k})R_{k}dxd\theta dz$$

(12) $$T=\frac{1}{2}\sum_{k=1}^{K}\int_{-h_{k}/2}^{h_{k}/2}\int_{0}^{\theta_{0}}\int_{0}^{L}\rho^{k}\left\{\begin{array}{l} {\left({\dot{u}}^{k}+f\frac{\partial \dot{w}}{\partial x}+g{\dot{\vartheta }}_{1}^{k}\right)}^{2}\\ +{\left[(1+\frac{z}{R_{i}}){\dot{v}}^{k}+f\frac{1}{R_{i}}\frac{\partial \dot{w}}{\partial \theta }+g{\dot{\vartheta }}_{2}^{k}\right]}^{2}+{\dot{w}}^{2} \end{array}\right\}R_{k}dxd\theta dz$$

where $\rho^{k}$ represents the density of *k*-th components.

The Lagrangian energy function can be written as

(13) $$\Pi =T_{\max}-U_{\max}$$

where $T_{\max}$ and $U_{\max}$ correspond to the maximum total kinetic energy and strain energy of the 3D RSOCCSs, respectively.

### 2.5. Admissible Displacement Functions and Solution

Within the Rayleigh–Ritz variational method, the selection of suitable admissible displacement functions is critical for achieving accurate results. In the present study, the displacement and rotation components of shell segments are generally expanded via the first-kind Chebyshev polynomial series, formulated as

(14) $$\begin{array}{l} \tilde{u}(x,\theta ,t)=U(x,\theta )e^{𝕀\omega *t}=\sum_{m=1}^{\infty }\sum_{1=1}^{\infty }A_{mn}\chi_{m}(x)\phi_{n}(\theta )e^{𝕀\omega *t}\\ \tilde{v}(x,\theta ,t)=V(x,\theta )e^{𝕀\omega *t}=\sum_{m=1}^{\infty }\sum_{n=1}^{\infty }B_{mn}\chi_{m}(x)\phi_{n}(\theta )e^{𝕀\omega *t}\\ \tilde{w}(x,\theta ,t)=W(x,\theta )e^{𝕀\omega *t}=\sum_{m=1}^{\infty }\sum_{1=1}^{\infty }C_{mn}\chi_{m}(x)\phi_{n}(\theta )e^{𝕀\omega *t}\\ \psi (x,\theta ,t)=\Psi (x,\theta )e^{𝕀\omega *t}=\sum_{m=1}^{\infty }\sum_{1=1}^{\infty }D_{mn}\chi_{m}(x)\phi_{n}(\theta )e^{𝕀\omega *t}\\ \varphi (x,\theta ,t)=\Phi (x,\theta )e^{𝕀\omega *t}=\sum_{m=1}^{\infty }\sum_{1=1}^{\infty }E_{mn}\chi_{m}(x)\phi_{n}(\theta )e^{𝕀\omega *t} \end{array}$$

where $A_{mn}$, $B_{mn}$, $C_{mn}$, $D_{mn}$ and $E_{mn}$ are corresponding Chebyshev expanded undetermined coefficient; $\chi_{m}(x)$ and $\phi_{n}(\theta )$ are, respectively, the *m*-th and *n*-th order Chebyshev polynomial for the displacements components in the x and θ directions, written as follows:

(15) $$\begin{array}{l} \chi_{1}(x)=1,\begin{array}{cc} & \chi_{2}(x)=x,\begin{array}{cc} & \chi_{m}(x)=2x\chi_{m-1}(x)-\chi_{m-2}(x)(m>2) \end{array} \end{array}\\ \phi_{1}(\theta )=1,\begin{array}{c} \begin{array}{cc} & \phi_{2}(\theta )=\theta ,\begin{array}{cc} & \phi_{m}(\theta )=2\theta \phi_{m-1}(\theta )-\phi_{m-2}(\theta )(m>2) \end{array} \end{array} \end{array} \end{array}$$

To assure completeness and orthogonality, it should be noted that the independent variable in the Chebyshev polynomial function must be specified inside the interval [−1, 1]. Therefore, The coordinate transformation from x∈[0,L] and $\theta \in [0,\theta_{0}]$ to $\bar{x}\in [-1,1]$ and $\bar{\theta }\in [-1,1]$ would be introduced into the present analysis. Owing to constraints on computer speed, capacity, and numerical accuracy, the proposed solution, despite its theoretical potential for arbitrary precision, requires truncating the polynomial terms to *M* and *N*. This truncation is implemented to simultaneously attain a satisfactory level of precision and computational efficiency. Free-free boundary conditions are enforced for the theoretical model: the bending moment and shear force at the axial ends (*x* = 0, *x* = *L*) and circumferential edges (*θ* = 0, *θ* = *θ*_0_) are zero. These conditions are satisfied by the Chebyshev polynomial-based displacement functions (Equations (14) and (15)), which inherently meet the zero-stress and zero-moment constraints at the boundaries.

The overall expression of the Lagrangian energy function with respect to the undetermined coefficients can be minimized via the Rayleigh–Ritz energy minimization approach.

(16) $$\frac{\partial 𝕃}{\partial \mathbb{R}}=0,\mathbb{R}=A_{mn},B_{mn},C_{mn},D_{mn},E_{mn}$$

Substituting the above equations into Equation (16) yields a matrix-form expression, which is given as follows:

(17) $$(K-\lambda_{mn}^{*}M)E_{mn}=0$$

where M denotes the mass matrix, K represents the stiffness matrix, and $E_{mn}$ stands for the eigenvectors. The frequencies along with their corresponding eigenvectors can be derived by solving Equation (17). Additionally, the circular frequency of the 3D RSOCCSs may be computed using Equation (18).

(18) ωmn=Re(λmn∗)

Notably, the theoretical model is tailored to the unique features of 3D RSOCCSs. Unlike traditional models for flat or closed shell structures, the kinematic relations (Equation (6)) and energy expressions (Equations (11) and (12)) explicitly incorporate the 3D reentrant core’s equivalent modulus (Equations (1)–(5)), which accounts for its negative Poisson’s ratio effect. This adaptation enables the model to capture the coupling between the core’s auxetic deformation and the shell’s curved vibration modes—an aspect overlooked in existing theoretical frameworks for auxetic structures.

## 3. Experiments

### 3.1. Specimen Design and Composite Structure Fabrication

In the current study, all 3D RSOCCSs specimens were fabricated with the following geometrical dimensions: $a_{1}=9.35\;\mathrm{mm}$, $a_{2}=a_{3}=9.00\;\mathrm{mm}$, $c_{1}=c_{2}=6.50\;\mathrm{mm}$, $\beta_{1}=\beta_{2}=\beta_{3}=60{}^{\circ}$, l=15.00 mm, t=b=1.00 mm, L=185.00 mm, H=20.00 mm, $R_{2}=80.00\;\mathrm{mm}$, which are graphically illustrated in Figure 1. For the fabrication of these 3D RSOCCSs composite specimens, two types of prepregs were employed: T700/epoxy carbon fiber plain-weave fabric prepreg and unidirectional carbon fiber/epoxy prepregs. These prepreg materials were supplied by Shanghai Kangzhan Composites Co., Ltd., located in Shanghai, China. Detailed information regarding the elastic properties of the aforementioned prepregs is provided in Table 1. The J-101 adhesive (used for core interlocking) exhibits a shear strength of ≥15 MPa after curing, while the J-272-A adhesive (used for face sheet-core bonding) has a shear strength of ≥25 MPa, ensuring compatibility with the CFRP laminates in service temperature ranges.

The fabrication of all-composite 3D reentrant auxetic-cored sandwich open circular cylindrical shells (3D RSOCCSs) involved a three-step process: laminate preparation via hot-press molding, 3D core assembly via interlocking and adhesive bonding, followed by integration with face sheets. This process was designed to ensure structural integrity and geometric precision of the 3D auxetic core, which is critical for its mechanical and vibration performance.

Step 1: Laminate Fabrication for Core Components and Face Sheets

CFRP laminates for both the 3D reentrant core components and inner/outer face sheets were fabricated using autoclave-based hot-press molding. The raw material was T700 carbon fiber/epoxy prepreg (supplied by Shanghai Kangzhan Composites Co., Ltd., Shanghai, China), chosen for its high specific strength and stiffness, typical in aerospace-grade composites.

① For core components: laminates with a symmetric ply configuration [(0°, 90°), 0°, 90°, 0°, 90°]_S_ were prepared. This layup was selected to balance in-plane stiffness in axial and circumferential directions, ensuring the core struts could withstand interlocking assembly forces.

② For face sheets: laminates with a ply sequence [0°, 90°, 0°] were used to enhance bending stiffness, as the face sheets bear primary bending loads during vibration.

③ Curing parameters: the autoclave process was conducted at 130 °C under a pressure of 0.5 MPa for 1.5 h, following the prepreg supplier’s specifications to ensure full curing of the epoxy matrix. Post-curing, laminates were cooled to room temperature at a rate of 2 °C/min to minimize residual stresses.

Step 2: 3D Core Assembly via Interlocking and Adhesive Bonding

**Figure 2:**
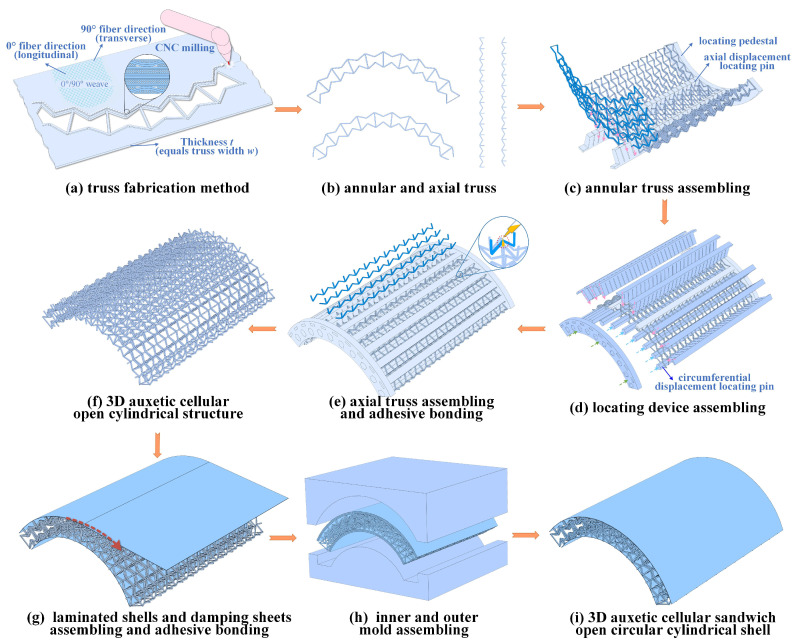
Manufacturing procedures of 3D RSOCCSs.

The 3D reentrant core (Figure 1c) consists of three types of 2D components: axial struts, circumferential rings, each with precision-machined interlocking grooves (Figure 2a,b).

① Machining: after curing, laminates were cut into these 2D components using CNC engraving (accuracy ±0.05 mm) to ensure the interlocking grooves (depth: 1.00 mm; width: 1.00 mm) matched perfectly, preventing gaps during assembly.

② Surface treatment: components were sanded with 400-grit sandpaper to create a rough surface (Ra ≈ 1.2 μm) and cleaned with acetone to remove contaminants, enhancing adhesive bonding.

③ Assembly and bonding: a custom positioning fixture (Figure 2c–e) was used to align axial struts and circumferential rings with diagonal reentrant struts, ensuring the designed reentrant angle and unit cell size. Liquid epoxy adhesive (J-101, Heilongjiang Petrochemical Research Institute, Harbin, China) was applied to the interlocking grooves (adhesive thickness ≈ 0.1 mm). The assembly was cured at 80 °C for 1 h under a clamping pressure of 0.1 MPa to avoid excessive adhesive squeeze-out.

Step 3: Integration of Core with Face Sheets

**Figure 3:**
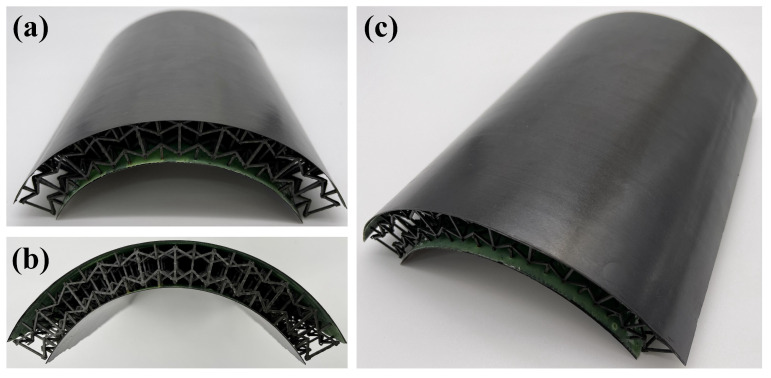
Photographic samples of 3D RSOCCSs.

The inner and outer face sheets (thickness: 1.00 mm) were bonded to the 3D core using J-272-A epoxy adhesive (Heilongjiang Petrochemical Research Institutee, Harbin, China), selected for its high shear strength (≥25 MPa) and compatibility with CFRP. A center support mold (Figure 2h) ensured uniform pressure (0.5 MPa) during bonding, preventing core deformation. Curing was performed at 130 °C for 1.5 h in an autoclave, consistent with the initial laminate curing cycle to avoid thermal degradation. Three replicate specimens were successfully produced for the baseline configuration (consistent with the geometric and material parameters above). The photographs of the successfully prepared specimens are presented in Figure 3.

The “hot-press molding + interlocking assembly” process developed here overcomes key challenges in fabricating all-composite 3D RSOCCSs. Unlike traditional bonding methods, the interlocking design ensures robust mechanical coupling, preserving the structural integrity required for accurate vibration testing. This fabrication strategy, validated by the consistency between experimental and numerical modal data (in Section 5), provides a reproducible protocol for producing complex auxetic-curved sandwich structures—critical for future experimental studies in this field.

### 3.2. Modal Hammer-Impact Testing

**Figure 4:**
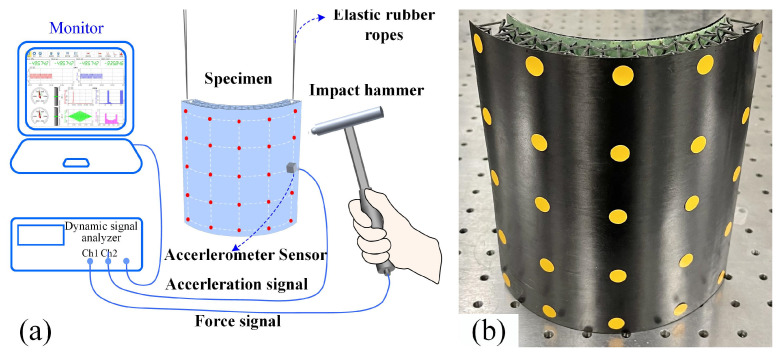
(**a**) Testing apparatus for modal hammer test, (**b**) signal measuring points in the actual specimen.

Modal hammer-impact testing (adopting a multi-point input and single-point output scheme) was conducted on the 3D RSOCCSs specimens to characterize their vibration behaviors, with a schematic illustration of the experimental apparatus provided in Figure 4. For each specimen, testing was performed across 25 evenly distributed grid points, which were arranged as 5 equal divisions in the θ-direction and 5 equal divisions in the x-direction. Prior to the modal tests, four flexible ropes were employed to suspend the specimen vertically, thereby simulating free-free boundary conditions, which is a setup critical for minimizing external constraints on the specimen’s natural vibration characteristics. The ropes were made of nylon (diameter 1 mm, length 500 mm) with an axial stiffness of 5 N/mm, ensuring minimal constraint on translational and rotational motions. During the testing process, the input signal was generated by sequentially impacting each calibration point individually with the hammer, while the output signal was measured at a fixed position using a small accelerometer. Specifically, an accelerometer (PCB SN46550, with a sensitivity of 10.07 mV/m/s^2^, Buffalo, NY, USA) and a force sensor (PCB SN30979, exhibiting a sensitivity of 12.25 mV/N, Buffalo, NY, USA) were utilized to simultaneously detect the acceleration response signal and the excitation force signal of the representative shell. Subsequently, the frequency response functions (FRFs) of the excitation and response signals were derived by applying a Fast Fourier Transform (FFT) to the collected and preprocessed data using a dynamic signal analyzer. For each specimen, repeat the modal hammering test 5 times at each of the 25 measurement points and take the average. Following this, through Multi-degree-of-freedom (MDOF) transfer function curve fitting, key modal parameters, including natural frequencies and modal shapes, were analyzed and extracted.

## 4. Finite Element Analysis Method

**Figure 5:**
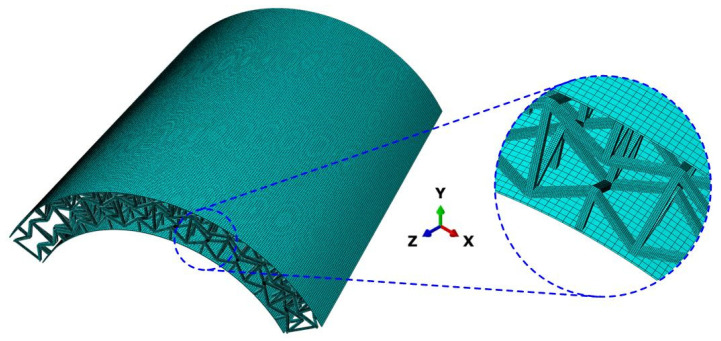
The FEA simulation model of 3D RSOCCSs.

In this section, to systematically investigate the vibration performance of 3D RSOCCSs, three-dimensional finite element models were established using ABAQUS 2019, following standard modal simulation procedures within the software. For the discretization of both the face sheets and 3D re-entrant auxetic cores, the C3D8R element was selected, this 8-node linear brick element with reduced integration is well-suited for such structural dynamics analyses due to its balance of computational efficiency and accuracy. In the modeling process, the outer face sheet, inner face sheet, and core were constructed with precise geometric fidelity, and their connections were defined through tie constraints; these constraints effectively simulate the perfect bonding condition between the face sheets and the core, mirroring the actual adhesive behavior in fabricated specimens. For the modal analysis setup, a linear perturbation “Frequency” step was configured in ABAQUS/Standard, with the “Lanczos” eigensolver employed to efficiently compute the natural frequencies and mode shapes, this solver is particularly advantageous for large-scale eigenvalue problems, ensuring rapid convergence. Prior to running the main simulation, a mesh convergence analysis was performed, confirming that mesh density would not affect the numerical results. The model was set to free-free boundary conditions (no displacement or rotation constraints) to match the experiment. Six rigid body modes (zero natural frequency) were explicitly identified in the FEA results, confirming the absence of artificial constraints. Upon completion of the analysis, the post-processing module in ABAQUS was utilized to extract key modal parameters, including structural natural frequencies and corresponding mode shapes. The detailed configuration of the 3D RSOCCSs finite element model, including mesh distribution and component connections, is illustrated in Figure 5.

## 5. Results and Discussion

### 5.1. Free Vibration Characterization

In this paper, a comprehensive comparative research of the first five-order modal characteristics of 3D RSOCCSs has been carried out. This research integrates three distinct yet complementary methodologies: the Rayleigh–Ritz theoretical analytical approach based on Reddy’s third-order shear deformation theory, the numerically finite element analysis (ABAQUS) technique, and the hammering modal test method. The synergistic use of these methods allows for a multi-faceted assessment of the modal behavior, ensuring both the rationality of the analysis and experimental verification.

**Figure 6:**
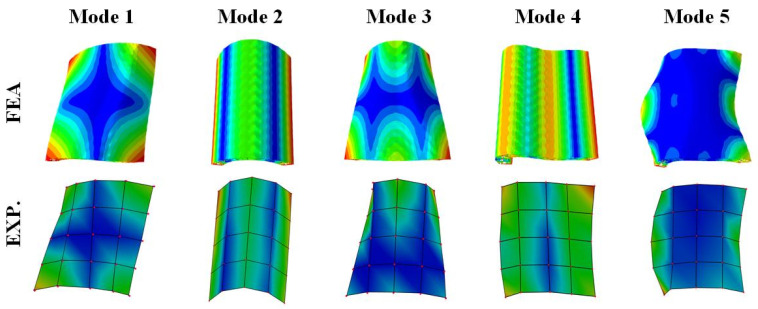
The first-five mode shapes of 3D RSOCCSs.

The modal shapes corresponding to the first five orders of the 3D RSOCCSs, derived from both experimental testing and numerical simulation, are presented in Figure 6. The comparison between these two sets of results reveals a remarkable degree of consistency, characterized by close agreement in both the spatial distribution and the fundamental patterns of the modal deformations. This high level of concordance serves as a strong validation of the finite element simulation methodology employed in this study, suggesting that the FEA approach is not only capable of accurately predicting the vibration modes of 3D RSOCCSs but also reliable for subsequent design analysis.

An in-depth investigation of the modal shape diagrams offers valuable insights into the underlying vibration mechanisms of 3D RSOCCSs. It is observed that the first five modal shapes are primarily constituted by a combination of bending vibration modes occurring along two principal directions: the axial direction of the sandwich open shell and its circumferential direction. Specifically, these modes can be categorized into distinct types, namely (1,1), (0,2), (1,2), (0,3), and (2,1), each representing a unique combination of axial and circumferential deformation patterns. The numerical notations here denote the number of half-waves in the axial and circumferential directions, respectively, providing a systematic way to characterize the complex modal behavior.

The cylindrical geometry of 3D RSOCCSs introduces several distinctive features to their modal characteristics. Unlike planar structures, which typically exhibit more straightforward deformation patterns, the curved nature of cylindrical shells leads to modal shapes that are inherently more complex and three-dimensional. The circumferential configuration of the cylindrical shell imposes significant constraints on the deformation behavior. The closed-loop nature of the circumferential direction makes it structurally stiffer compared to the axial direction, which is open-ended. As a result, 3D RSOCCSs demonstrate a pronounced tendency to undergo bending deformations preferentially in the axial direction, while resisting deformation in the circumferential direction. This directional dependence of the bending behavior is a direct consequence of the geometric and structural characteristics of 3D RSOCCSs and has important implications for their dynamic performance in practical applications.

**Figure 7:**
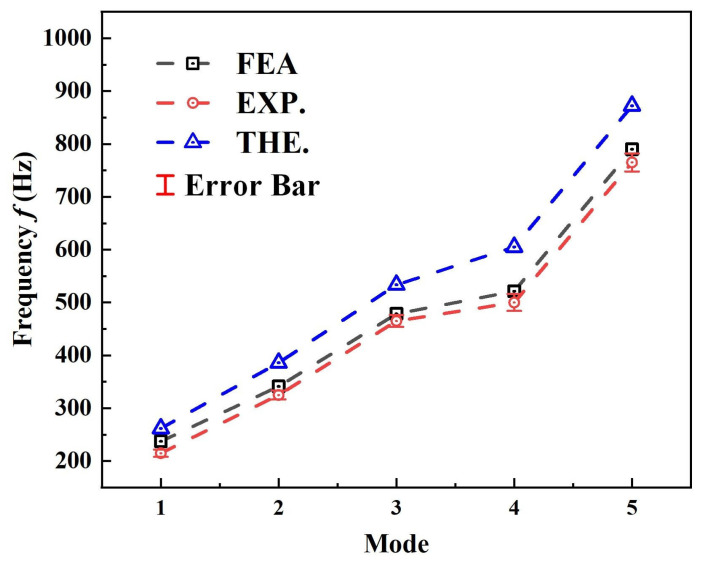
The first-five mode natural frequency of 3D RSOCCSs in free vibration.

The results of the first-five order natural frequencies obtained from theoretical analysis, experimental testing, and finite element simulation are presented in Figure 7. By virtue of its comprehensive consideration of the intricate geometry of the negative Poisson’s ratio sandwich core in 3D RSOCCSs, a comparative analysis of these results reveals that the finite element method yields prediction results that are in close agreement with experimental data, with an average error of no more than 10%. The discrepancies observed are primarily attributed to imperfections introduced during the manufacturing process and inherent errors associated with experimental measurements. The theoretical calculations, which necessitate the equivalence of the complex three-dimensional reentrant honeycomb negative Poisson’s ratio sandwich core and inevitably involve certain methodological simplifications, exhibit a slightly larger deviation from the experimental results. Nevertheless, the average error remains within an acceptable range of less than 20%.

Based on the above, both the simulation prediction model established using finite element software (ABAQUS 2019) and the theoretical prediction model based on the Rayleigh–Ritz method demonstrate efficacy in analyzing the free vibration characteristics of 3D RSOCCSs. The finite element approach, with its ability to capture the detailed geometric and material properties of the structure, provides a high level of accuracy, while the theoretical method offers fast and convenient calculations with a certain degree of accuracy. Therefore, these complementary methods contribute to a comprehensive understanding of the vibration properties of 3D RSOCCSs.

Notably, this triple-method verification (theoretical, FEA, experimental) represents a departure from existing studies on auxetic structures, which typically rely on single-method analysis or simplify the core as a homogenized layer. By validating the theoretical model against both high-fidelity 3D FEA (with cell-level discretization, Figure 5) and experiments on physically fabricated specimens, a reliable framework was established for quantifying vibration in 3D auxetic-curved systems.

### 5.2. Influence of the Fiber Ply Angle

**Figure 8:**
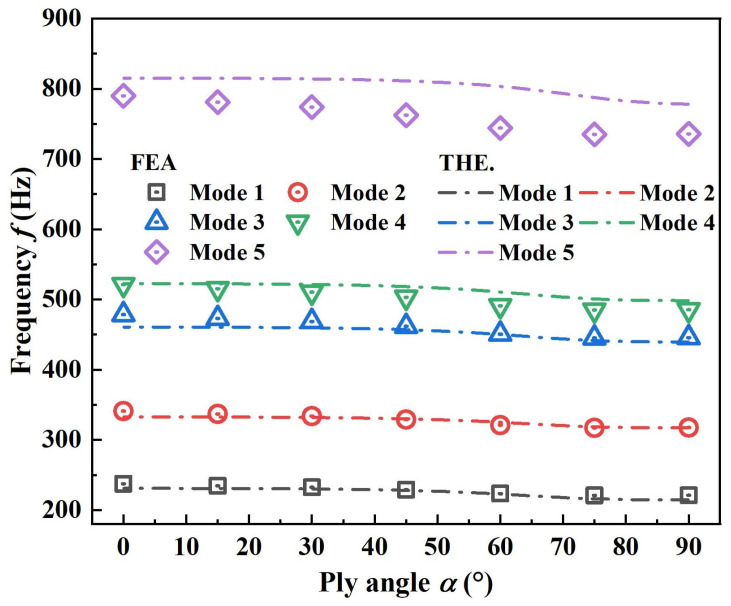
Influence of composite material fiber laying angle α on natural frequency *f* of 3D RSOCCSs in free vibration.

In the field of composite material design, the fiber angle stands out as a crucial parameter, primarily determined by the performance characteristics of composite materials. A prominent feature of composite materials is their anisotropy, meaning their properties vary significantly with changes in the fiber direction. By rationally adjusting the fiber angle, it is possible to precisely regulate important mechanical properties of composite materials such as stiffness, strength, and stability. Therefore, in this section, in order to investigate the influence of fiber ply angles on free vibration performance, a systematic study was conducted on the inner and outer curved shells with unidirectional ply angles. The variation in the first five natural frequencies with the unidirectional ply angle is shown in Figure 8. Both theoretical and finite element prediction results indicate that the natural frequencies of 3D RSOCCSs gradually decrease as the value of the ply angle becomes smaller, changing gently from 0° to 50° and more dramatically from 50° to 90°. The degree of influence gradually increases with the increase in the vibration order, having a greater impact on high-order vibrations. This is mainly because the fiber angle directly determines the in-plane stiffness (such as tensile and bending stiffness) of the composite material. When the fiber direction is consistent with the main vibration stress direction, the overall structural stiffness increases, leading to a higher natural frequency; conversely, a deviation in the angle reduces the stiffness, resulting in a decrease in the natural frequency. For example, a cylindrical shell with fibers laid along the circumferential direction has a significantly higher radial vibration frequency than a structure with obliquely laid fibers. Alterations in the fiber angle can modify the directional distribution of structural stiffness, leading to shifts in the energy distribution among different modal shapes (such as bending, torsion, and axial vibration). Specific fiber angle combinations can suppress vibration amplitudes in particular directions. The observed trend—natural frequencies decreasing with increasing fiber angle, particularly for high-order modes—differs from findings in closed cylindrical shells or flat auxetic panels. In closed shells, circumferential stiffness dominates, making fiber angle effects less sensitive to mode order; however, in open 3D RSOCCSs, the open boundary weakens circumferential constraint, amplifying the influence of axial stiffness (regulated by fiber angle) on high-order axial-bending modes. This distinction highlights the need for tailored design strategies for open auxetic-curved structures.

### 5.3. Influence of Geometric Parameters

**Figure 9:**
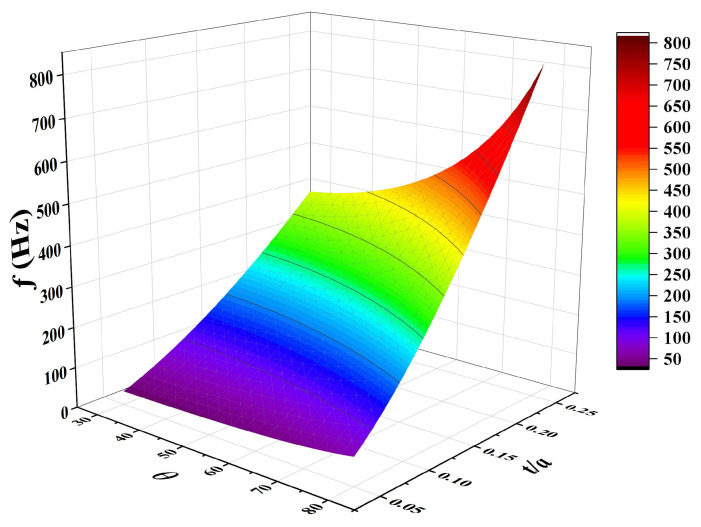
Influence of geometric design parameters *t*/*a*, β on natural frequencies *f* of 3D RSOCCSs in free vibration.

As an important part of the sandwich structure, the core significantly improves the bending stiffness and shear strength of the structure by bearing the shear force and the supporting panel, and at the same time reduces the overall mass with the light weight characteristics to achieve the balance of “high stiffness and light weight”. The material and configuration of the core directly affect the dynamic characteristics of the structure. The rich design parameters (such as honeycomb size, concave angle, wall thickness, etc.) of the three-dimensional concave honeycomb negative Poisson’s ratio structure can flexibly adjust its stiffness, mass distribution and Poisson’s ratio characteristics, so as to accurately optimize the vibration performance. Through parameter adjustment, the natural frequency, mode shape of the structure can be changed to achieve resonance avoidance or vibration suppression. Therefore, it is necessary to analyze the influence of core geometry design parameters of 3D RSOCCSs on their modal performance. In this section, based on the Rayleigh–Ritz theoretical method, the effects of the main design parameters of the three-dimensional concave honeycomb negative Poisson’s ratio structure on the natural frequency of 3D RSOCCSs are analyzed, and the relevant analytical results are shown in Figure 9.

**Figure 10:**
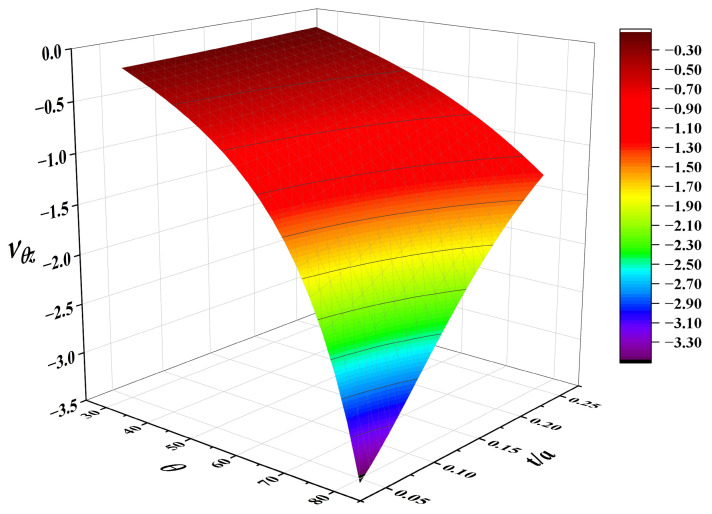
Influence of geometric design parameters *t*/*a*, β on Poisson’s ratios $\nu_{\theta z}$ of 3D RSOCCSs in free vibration.

Under the premise that the core thickness of the sandwich structure remains unchanged, the natural frequencies of 3D RSOCCSs increase with the increase in the re-entrant angle and slenderness ratio. It can be easily derived from the formulas in Section 2.2 that this is mainly due to the significant increase in the stiffness of the 3D reentrant honeycomb negative Poisson’s ratio core caused by the increase in these two parameters (re-entrant angle and slenderness ratio). As the core supporting layer of the sandwich structure, the increase in core stiffness can significantly improve the overall bending and shear stiffness of the structure. According to vibration theory, the natural frequency of a structure is proportional to the square root of its stiffness; thus, the increase in core stiffness directly leads to an increase in the overall natural frequency. From the analysis of Figure 10, it can be concluded that there is a negative correlation between the negative Poisson’s ratio effect and the natural frequency characteristics of the 3D RSOCCSs structure in the parameter-performance design. That is, the vibration characteristics of 3D RSOCCSs can be qualitatively evaluated by predicting the negative Poisson’s ratio value. Compared with the complex calculation of vibration analysis, the negative Poisson’s ratio effect of the structure can be easily obtained by geometric analysis, which provides convenience for the modal performance evaluation of complex three-dimensional surface structures. Based on the above analysis, in the vibration scene application of A of 3D RSOCCSs, the spatial distribution of structural stiffness can be optimized by increasing the core stiffness, which balances the stiffness contribution of each mode, reduces low-frequency resonance points caused by local low-stiffness regions, and enhances the frequency interval of high-order modes to avoid mode clustering. The inverse correlation between NPR magnitude and natural frequency (Figure 10) reveals a novel tuning mechanism: increasing the re-entrant angle reduces NPR (making it less negative) while enhancing core stiffness, thereby raising natural frequencies. This contrasts with 2D auxetic cores, where NPR and stiffness are often coupled linearly. For 3D RSOCCSs, this nonlinear relationship enables multi-objective optimization (e.g., balancing vibration suppression and auxetic energy absorption)—a critical insight for engineering applications like aerospace components.

## 6. Conclusions

This paper advances the literature through three key contributions: (1) it is the first to characterize the free vibration behavior of 3D reentrant auxetic core-reinforced CFRP sandwich open cylindrical shells (3D RSOCCSs), filling gaps in research on complex auxetic-curved structures; (2) it establishes a validated multi-method framework (theoretical, FEA, experimental) for analyzing such systems, overcoming limitations of simplified models; (3) it reveals novel design methods, including fiber angle effects unique to open shells and synergistic tuning of frequency and negative Poisson’s ratio (NPR) via core geometry.

To achieve these, this work employed a comprehensive methodological approach: an analytical model for 3D RSOCCSs’ modal characteristics was developed based on the Rayleigh–Ritz method and Reddy’s third-order shear deformation theory; a finite element simulation model was constructed using ABAQUS; and all-composite 3D RSOCCSs specimens were fabricated via hot-press molding and interlocking assembly, with their modal parameters tested via the modal hammering method. Experimental results confirmed good agreement between analytical/simulation predictions and test data, validating the models’ effectiveness in predicting free vibration characteristics of 3D RSOCCSs. Further analysis of design parameters revealed that: (1) the natural frequencies of 3D RSOCCSs decrease with increasing fiber laying angle of continuous fiber-reinforced composites, primarily due to the angle’s influence on the bending stiffness of inner and outer curved shell panels; (2) geometric parameters of the 3D reentrant honeycomb core significantly affect natural frequencies, enabling regulation of free vibration and NPR characteristics through rational parameter design.

These findings hold substantial practical and theoretical value. Practically, they provide critical engineering guidance: in aerospace and marine fields, frequency tuning via fiber angles and core geometry helps avoid resonance in components like rocket fairing segments and submersible pressure hulls (mitigating vibration-induced fatigue); the all-composite design (CFRP face sheets + CFRP auxetic core) addresses corrosion issues of metal–auxetic hybrids, suiting humid/marine environments; validated theoretical and FEA models offer efficient tools for predicting vibration behavior, accelerating industrial adoption of auxetic-cored shells in lightweight applications. Theoretically, the study expands multifunctional application scenarios of NPR metamaterials and provides reference for vibration control design based on composite sandwich cylindrical shells.

Future work could be focused on (1) extending to nonlinear vibration analysis under large-amplitude excitation or blast loads to address geometric and material nonlinearity; (2) investigating multiphysics coupling (vibration–temperature–moisture) for aerospace/marine service conditions; (3) topology optimization of 3D reentrant cores to target specific frequency ranges or damping performance; (4) studying dynamic failure mechanisms (e.g., interfacial debonding under cyclic vibration) to ensure long-term reliability; and (5) scaling to industrial-sized specimens (1~2 m) to validate size effects and practical manufacturability.

## Figures and Tables

**Table 1:** The mechanical properties of the composite materials.

Property	Symbol	Unidirectional	Plain-Weave
Young’s modulus	$E_{11}$	122 GPa	48.4 GPa
	$E_{22}$	8.5 GPa	48.4 GPa
Poisson’s ratio	$\nu_{12}$	0.28	0.3
	$\nu_{23}$	0.28	0.3
Shear modulus	$G_{12}$	4 GPa	4 GPa
	$G_{23}$	3 GPa	3 GPa

## Appendix A

(A1)
$$K_{s}^{a}=E_{s}bt/a$$

(A2)
$$K_{s}^{l}=E_{s}bt/l$$

(A3)
$$K_{f}^{a}=E_{s}bt^{3}/a^{3}$$

(A4)
$$\Theta (K_{i},\beta )=\frac{K_{f}^{a}K_{s}^{a}\cos\beta }{16K_{s}^{l}(K_{s}^{a}\cos^{2}\beta +K_{f}^{a}\sin^{2}\beta )}$$

(A5)
$$\Theta^{\prime }=\frac{\tan\beta (K_{f}^{a}K_{s}^{a}+K_{f}^{a}K_{s}^{l})}{\tan\beta \sin\beta (K_{f}^{a}K_{s}^{a}+K_{f}^{a}K_{s}^{l})+\cos\beta (K_{f}^{a}K_{s}^{a}+2K_{s}^{a}K_{s}^{l})}$$
